# MicroGMT: A Mutation Tracker for SARS-CoV-2 and Other Microbial Genome Sequences

**DOI:** 10.3389/fmicb.2020.01502

**Published:** 2020-06-25

**Authors:** Yue Xing, Xiao Li, Xiang Gao, Qunfeng Dong

**Affiliations:** ^1^Department of Veterinary Integrative Biosciences, Texas A&M University, College Station, TX, United States; ^2^Department of Molecular and Cellular Medicine, Texas A&M University, College Station, TX, United States; ^3^Department of Medicine, Stritch School of Medicine, Loyola University Chicago, Maywood, IL, United States; ^4^Center for Biomedical Informatics, Stritch School of Medicine, Loyola University Chicago, Maywood, IL, United States

**Keywords:** COVID-19, SARS-CoV-2, molecular evolution, epidemiology, bioinformatics, mutation, microbial, virus

## Abstract

With the continued spread of SARS-CoV-2 virus around the world, researchers often need to quickly identify novel mutations in newly sequenced SARS-CoV-2 genomes for studying the molecular evolution and epidemiology of the virus. We have developed a Python package, MicroGMT, which takes either raw sequence reads or assembled genome sequences as input and compares against database sequences to identify and characterize indels and point mutations. Although our default setting is optimized for SARS-CoV-2 virus, the package can be also applied to any other microbial genomes. The software is freely available at Github URL https://github.com/qunfengdong/MicroGMT.

## Introduction

Since the first submission of severe acute respiratory syndrome coronavirus 2 (SARS-CoV-2) genome sequence to GISAID on January 10th, 2020, there have already accumulated 34786 sequences at GISAID by May 31st, 2020 (Elbe and Buckland-Merrett, 2017; Shu and McCauley, 2017). The genome sequences of SARS-CoV-2 will continue accumulating since researchers heavily rely on DNA sequencing to study the epidemiology and molecular evolution of the virus (Lanciotti et al., 1994; Chinese SARS Molecular Epidemiology Consortium, 2004; Faye et al., 2014). SARS-CoV-2 is an RNA virus with limited proofreading capability of correcting replication errors (Domingo and Holland, 1997; Domingo et al., 2008; Duffy et al., 2008; Denison et al., 2011), thus it has been evolving continuously with new mutations (Bar-On et al., 2020; Koyama et al., 2020; Pachetti et al., 2020), which inevitably face selective pressures imposed by the host. Although most mutations are expected to be selectively neural, it is important to monitor if SARS-CoV-2 will eventually evolve to be a stronger or weaker infectious agent as time goes on. Therefore, it is vital to track mutations from newly sequenced SARS-CoV-2 genome.

Although whole genome sequencing of SARS-CoV-2 can be performed rapidly these days, bioinformatics tools are required to keep pace with the rapid sequencing capacity for tracking the mutations from newly sequenced genomes. To the best of our knowledge, there currently only exist two bioinformatics tools publicly available for detecting SARS-CoV2 mutations. The first is a web-based platform recently released by the Los Alamos National Laboratory (Korber et al., 2020). However, the tool is only limited for characterizing known mutations for the SARS-CoV-2 in genic regions. Users cannot submit their own sequences (e.g., newly sequenced viral genome isolated from COVID-19 patients) to compare with existing mutations in the database and identify new mutations. The second, named CoV-Glue, is also a web-based platform^1^. At the time of our manuscript preparation, CoV-Glue is still undergoing development and testing before its official release according to its web site. The main limitation with the current version of CoV-Glue is that it cannot detect mutations among multiple input sequences provided by users (only mutations between input and database sequences can be identified). In addition, both the above web-based tools can only analyze SARS-CoV-2 sequences instead of other microbial species. Therefore, we have developed a command-line based Python package, Microbial Genomics Mutant Tracker (MicroGMT), for users to detect mutations from their own sequence data (either for SARS-CoV-2 or other microbial genomes).

## Methods

### Implementation

**FIGURE 1:**
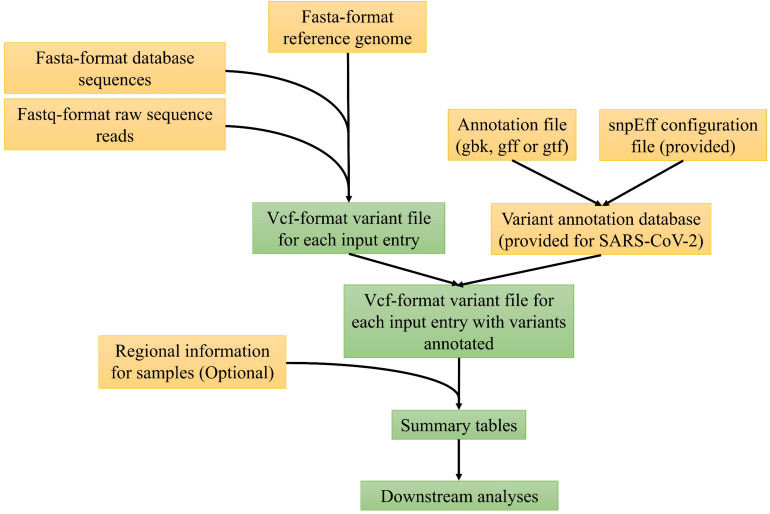
The overview of MicroGMT workflow. See main text for details.

Figure 1 depicts the major computational steps of MicroGMT for either any microbial species or SARA-CoV-2. The required inputs to MicroGMT are (1) a fasta-format reference genome sequence, (2) a Genbank file, GTF or GFF-format annotation file for the reference genome, and (3) the user’s own query sequences either as fastq-format raw sequence reads or fasta-format assembled genome sequences. The default reference genome of SARA-CoV-2 and its genome annotation are already included in the package [i.e., SARA-CoV-2 isolate Wuhan-Hu-1 complete genome sequence with GenBank accession number NC_045512 (Wu et al., 2020)]. We chose to perform pairwise alignment instead of multiple sequence alignment for two reasons: (1) biologically, researchers are mainly interested in using the reference genome as the coordinates to refer to the mutation. MicroGMT infers any genomic difference among non-reference genomes based on their individual alignment to the reference genome (i.e., do they carry the same or different mutation in a given genomic position with respect to the reference genome); (2) computationally, we can easily scale up the pairwise alignment by only computing each of new additional sequences instead of re-computing the entire multiple sequence alignment. This is an important computational issue to consider as there are already 34786 SARS-CoV-2 sequences in the database as of May 31st, 2020 and we expect that the number of database sequences will continue to grow rapidly. For SARS-CoV-2, we have already performed all the pairwise alignment for all the existing database sequences against the reference sequence and the results are already stored in pre-built summary tables as part of our package. When users download all the latest SARS-CoV-2 genome sequences (e.g., from GISAID.org), our package provides a utility script to compare their downloaded version with the precomputed ones in our package and perform the pairwise alignment only with the new ones. The BAM-format alignment outputs produced by minimap2 (Li, 2018) are used as input for the subsequent identification of genomic variants with bcftools (Li, 2011). If necessary, users can set the prior probability for the identification of variants. For user-supplied raw sequence reads, MicroGMT aligns them to the reference genome by the standard procedure consisting of BWA (Li and Durbin, 2009), Samtools (Li et al., 2009), and Picard^2^ which performs alignment, sorting, duplication removal, and indel realignment for both single-end or paired-end sequence reads. For the aligned reads, GATK (McKenna et al., 2010; Van der Auwera et al., 2013) is used by MicroGMT for variant calling. Users can choose the cut-off of mapping quality and base quality for variant calling. All the identified mutations are listed in vcf-format outputs, to be characterized by snpEff (Cingolani et al., 2012) at both the DNA and protein levels for each mutation.

Based on the above results, MicroGMT automatically generates mutation summary tables, in which each identified mutation is further characterized as follows: (1) where is the genomic location of the mutation (e.g., genic or intergenic regions), (2) what is the mutation in the genomic and protein sequence (e.g., synonymous or non-synonymous), and (3) is it a novel mutation (i.e., never been identified in other existing genome sequences), or which/how many other existing genome sequences carry it already. If the optional regional file is provided by the user, this information is also incorporated in the summary tables: the software searches the sequence/sample ID in the provided file to extract regional information for the respective vcf-format output, and adds regional information to the sequence/sample ID in the table header. Using the summary tables, users can easily look at specific genes of interest by filtering the “gene” column of the summary tables. Users can also restrict their query within a specific genomic region of interest. For example, they may ask if there is any mutation in their supplied sequences within the region of Spike gene by restricting their query for the genomic coordinates of the Spike gene. MicroGMT was built under Python 3 and can be used in Linux/Mac OS systems.

### Validation

We simulated mutations in 1000 SARS-CoV-2 genome sequences and their corresponding raw sequence reads to validate the performance of MicroGMT. The complete genome sequence of SARS-CoV-2 isolate Wuhan-Hu-1 (GenBank accession number NC_045512) was used as the reference genome for simulating mutations. The simuG program (Yue and Liti, 2019) was used to simulate random point mutations and indels to the reference genome sequence. The number of mutations in each simulated genome sequence was drawn from a Poisson distribution based on the average number of mutations per genome that we observed among real SARS-CoV-2 sequences in GISAID. Using those simulated genome sequences, their corresponding raw sequence reads were also simulated using ART-illumina (Huang et al., 2012) with the following parameters: sequencing platform of HiSeq2500, fold coverage of 50, paired-end read length of 150 bp, average fragment size of 300 bp with standard deviation of 20 bp, and base quality between 18 and 38 (Supplementary File 1). For evaluating the validation results, sensitivity is defined as the number of genomic mutations identified by MicroGMT divided by the number of true mutations introduced via simulation; specificity is defined as the number of genomic loci without mutations identified by MicroGMT divided by the number of genomic loci without any simulated mutations; accuracy is defined as the sum of genomic loci with and without mutations identified by MicroGMT divided by the total number of genomic loci with and without simulated mutations.

## Results

To validate the reliability of MicroGMT for mutation identification, 1000 artificial mutant strains of SARS-CoV-2 were simulated in which point mutations and/or indels were introduced randomly based on a Poisson distribution with the observed average number of mutations per genome in the real dataset of GISAID. By applying MicroGMT to this simulated test dataset, we obtained a specificity of 100%, a sensitivity of 100%, and an accuracy of 100% for both simulated whole genome sequences and raw sequence reads. In other words, MicroGMT is highly reliable in correctly identifying all mutations.

For real-world SARS-CoV-2 sequences, we would not know the exact genomic position of each potential mutation as in the simulated dataset. Therefore, we evaluated the reliability of MicroGMT through random sampling and manual verification. Specifically, we applied MicroGMT to ten randomly selected SARS-CoV-2 whole genome sequences, as well as their simulated paired-end raw sequence reads using ART-illumina (fold coverage of 10, read length of 150 bp, average fragment size of 300 bp with standard deviation of 20 bp) as test samples. Sensitivity and accuracy were both 100% for whole genome sequences, and were 98.4 and 100% for simulated raw sequence reads, respectively. The only point mutation that was not identified by MicroGMT for raw sequence reads was only 28 base pairs away from a stretch of unassigned bases (Ns), making it difficult to align the raw reads at that region (a problem with quality of the raw reads).

To illustrate the utility of MicroGMT, we applied it to identify all the mutations in a total of 34786 strains of SARS-CoV-2 in the GISAID database as of May 31st, 2020. In total, 15312 unique mutations were identified from 12618 genomic loci in SARS-CoV-2 reference genome (14749 mutations were located inside genes at 12245 loci). The running time of MicroGMT scales linearly with the number of input sequences. It took roughly 40 h to process all 34786 sequences. For a user with 100 input sequences, it would take less than 7 min for assembled genome sequences and less than 30 min for raw sequence reads with 50x depth of coverage.

## Discussion

MicroGMT can identify, annotate and summarize mutations in a large number of microbial genome sequences. Although it is optimized for SARS-CoV-2 sequences, it can be easily applied to other microbial sequences (potentially any haploid genomes). By tracking mutations among closely related strains, MicroGMT identifies where and what kinds of the mutations are at both DNA and protein levels.

MicroGMT is designed for tracking indels and SNPs among closely related strains instead of detecting large-scale complex genomic rearrangements and duplications. In addition, if users supply fastq-format raw sequence reads as input, the accuracy of mutation detection can be slightly affected by unmasked repetitive regions in the reference genome (results not shown) due to the difficult nature of aligning short sequence reads to the reference genome.

Overall, MicroGMT provides a fast, flexible, and reliable solution for researchers who need to track genomic mutations for SARS-CoV-2 virus or other microbial species.

## Data Availability Statement

Publicly available datasets were analyzed in this study. The datasets analyzed for this study is available at the GISAID website at https://www.gisaid.org/. The MicroGMT software is freely available at https://github.com/qunfengdong/MicroGMT.

## Author Contributions

YX implemented the software and drafted the manuscript. XL and YX performed the testing and data analysis. XG and QD conceived the project and design. All authors contributed to the article and approved the submitted version.

## Conflict of Interest

The authors declare that the research was conducted in the absence of any commercial or financial relationships that could be construed as a potential conflict of interest.

## Footnotes

1 http://cov-glue.cvr.gla.ac.uk/

2 https://broadinstitute.github.io/picard/
