# Supplementary material for: MicroGMT: A Mutation Tracker for SARS-CoV-2 and Other Microbial Genome Sequences
Source: Front Microbiol. 2020 Jun 25;11:1502. doi: 10.3389/fmicb.2020.01502 (PMC7330013; doi:10.3389/fmicb.2020.01502)
Supplement: Supplementary file 1 [file Data_Sheet_1.PDF]

Supplemental File 1

Simulation of 1000 SARS-CoV-2 strains

Determine number of mutations per strain

(python 3.6)

```
import random
import os
import math
import sys
import numpy

def draw_random_poisson(n,lam,in_zero):
    i=0
    mean_vec=[]
    if in_zero==False:
        while i < n:
            a=0
            while a==0:
                a=numpy.random.poisson(lam)
            mean_vec.append(str(a))
            i += 1
    else:
        while i < n:
            a=numpy.random.poisson(lam)
            # 0-2 only
            if a>2:
                a=0
            mean_vec.append(str(a))
            i += 1
    return mean_vec

in_n=1000

out_snp_table="snp_number.txt"
out_ind_table="ind_number.txt"

all_snp_num=draw_random_poisson(in_n,3,False)
all_ind_num=draw_random_poisson(in_n,1,True)

with open(out_snp_table,'w') as f:
    f.write("\n".join(all_snp_num))

f.close()

with open(out_ind_table,'w') as f:
    f.write("\n".join(all_ind_num))

f.close()
```

Simulate genome sequences with mutations

(bash)

Genome assembly sequences

```
for i in {1..1000}
do
a=$(sed -n "${i},${i}p" snp_number.txt)
b=$(sed -n "${i},${i}p" ind_number.txt)
perl /scratch/user/inumia/simuG/simuG.pl \
-r NC_045512.fa \
-snp_count $a -indel_count $b \
-p test${i}
done

for i in {1..1000}
do
    rm -f tmp
    grep -v "^#" test${i}.refseq2simseq.INDEL.vcf > tmp
    cat test${i}.refseq2simseq.SNP.vcf tmp > test${i}.ref.vcf
done

mkdir fa
mv *simseq.genome.fa fa

mkdir refvcf
mv test*.ref.vcf refvcf

for i in {1..1000}
do
    sed -i '1d' fa/test${i}.simseq.genome.fa
    sed -i "1 i >test${i}" fa/test${i}.simseq.genome.fa
done

cat fa/* > sequences.fasta
```

Corresponding raw reads sequences

```
cd /scratch/user/inumia/art_bin_MountRainier
for i in {1..1000}
```

```
do
./art_illumina \
-i /scratch/user/inumia/test/simulation/fa/test${i}.simseq.genome.fa \
-ss HS25 -p -l 150 -f 50 --mflen 300 -s 20 -na \
-qL 18 -qU 38 \
-o /scratch/user/inumia/test/simulation/fq/test${i}_
done

ls fq > fq.list
sed -i 's/_1.fq//g' fq.list
sed -i 's/_2.fq//g' fq.list
sort fq.list | uniq > fq.list2
rm -f fq.list
mv fq.list2 fq.list
```

Annotate ref vcfs

```
python /scratch/user/inumia/MicroGMT/annotate_vcf.py \
-i refvcf -o refvcf/out2 \
-f b \
-eff /sw/eb/software/snpEff/4.3t-foss-2018b-Python-3.6.6-Java-1.8.0
```

Call variants by MicroGMT

Genome assembly sequences

```
python /scratch/user/inumia/MicroGMT/sequence_to_vcf.py \
-r /scratch/user/inumia/MicroGMT/NC_045512_source_files/NC_045512.fa \
-i assembly -fs sequences.fasta \
-o faout

python /scratch/user/inumia/MicroGMT/annotate_vcf.py \
-i faout -o faout/out2 \
-f b \
-eff /sw/eb/software/snpEff/4.3t-foss-2018b-Python-3.6.6-Java-1.8.0
```

Raw reads sequences

```
cat fq.list | while read line
do
  python /scratch/user/inumia/MicroGMT/sequence_to_vcf.py \
  -r /scratch/user/inumia/MicroGMT/NC_045512_source_files/NC_045512.fa \
  -i fastq -fq1 fq/${line}_1.fq -fq2 fq/${line}_2.fq \
  -o fqout \
  -gatk $EBROOTGATK \
  -picard $EBROOTPICARD \
  -l ${line}.log -n ${line} -ki
done

python /scratch/user/inumia/MicroGMT/annotate_vcf.py \
-i fqout -o fqout/out2 \
-f b \
-eff /sw/eb/software/snpEff/4.3t-foss-2018b-Python-3.6.6-Java-1.8.0
```

Find concordance with NC_045512

Genome assembly sequences

```
for i in {1..1000}
do
cat refvcf/test${i}.ref.vcf | \
awk '$1 ~ /^#/ {print $0;next} {print $0 | "sort -k1,1 -k2,2n"}' \
> sort_refvcf/test${i}.ref.vcf
done
```

Raw reads sequences

```
for i in {1..1000}
do
java -jar /sw/eb/software/snpEff/4.3t-foss-2018b-Python-3.6.6-Java-1.8.0/SnpSift.jar \
concordance -v sort_refvcf/test${i}.ref.vcf faout/test${i}.vcf
done
```
